# Capillary malformations in unusual territory: Klippel–Trénaunay syndrome with penoscrotal involvement

**DOI:** 10.1093/skinhd/vzaf103

**Published:** 2026-01-09

**Authors:** Mahesh Mathur, Sumit Paudel, Nabita Bhattarai, Sandhya Regmi, Himanshu Pathak, Sambidha Karki

**Affiliations:** Department of Dermatology, College of Medical Sciences, Bharatpur, Nepal; Department of Dermatology, College of Medical Sciences, Bharatpur, Nepal; Department of Dermatology, College of Medical Sciences, Bharatpur, Nepal; Department of Dermatology, College of Medical Sciences, Bharatpur, Nepal; Department of Dermatology, College of Medical Sciences, Bharatpur, Nepal; Department of Dermatology, College of Medical Sciences, Bharatpur, Nepal

## Abstract

Klippel–Trénaunay syndrome (KTS) is a rare congenital vascular disorder that typically presents with capillary and venous malformations, limb hypertrophy and, in some cases, lymphatic abnormalities. We report a 3-year-old boy with extensive vascular malformations affecting the left lower limb, predominantly involving the penoscrotal region – an uncommon presentation without systemic involvement. This case illustrates the diagnostic complexity of genitourinary KTS and emphasizes the need for vigilant monitoring, multidisciplinary input and tailored management to reduce complications and improve long-term outcomes.

## Teaser

We present a rare case of Klippel-Trenaunay Syndrome with extensive penoscrotal involvement in a child. Despite diffuse genital and cutaneous disease, there was no visceral extension, supporting a favourable prognosis and emphasizing the need for multidisciplinary follow-up.

What is already known about this topic?

- Klippel–Trénaunay syndrome (KTS) is a rare congenital condition characterized by capillary malformation, venous malformation and soft tissue or bone hypertrophy, typically affecting the lower limb.
- Genital involvement is uncommon but clinically significant in KTS.

What does this study add?

- Extensive penoscrotal involvement without visceral disease indicates a more favourable prognosis in patients with KTS.
- Through this case, we highlight the importance of multidisciplinary care with long-term surveillance in patients with KTS.

Klippel–Trénaunay syndrome (KTS) is an uncommon congenital capillary–lymphatic–venous malformation with an estimated incidence of approximately 1 in 100 000 live births.^1^ It is characterized by a classic triad of capillary malformation (also known as port wine stain), venous malformation and soft tissue or bone hypertrophy, with or without lymphatic abnormalities.^2^ Involvement of the lower limb is common; however, diffuse cutaneous and genital involvement in young children is rare and poses significant functional, ­psychosocial and management challenges.^2^ We hereby present a case of a 3-year-old child diagnosed with KTS with extensive vascular malformations involving the penoscrotal region and left lower extremity, highlighting clinical features, imaging findings and management challenges.

## Case report

**Figure 1.**
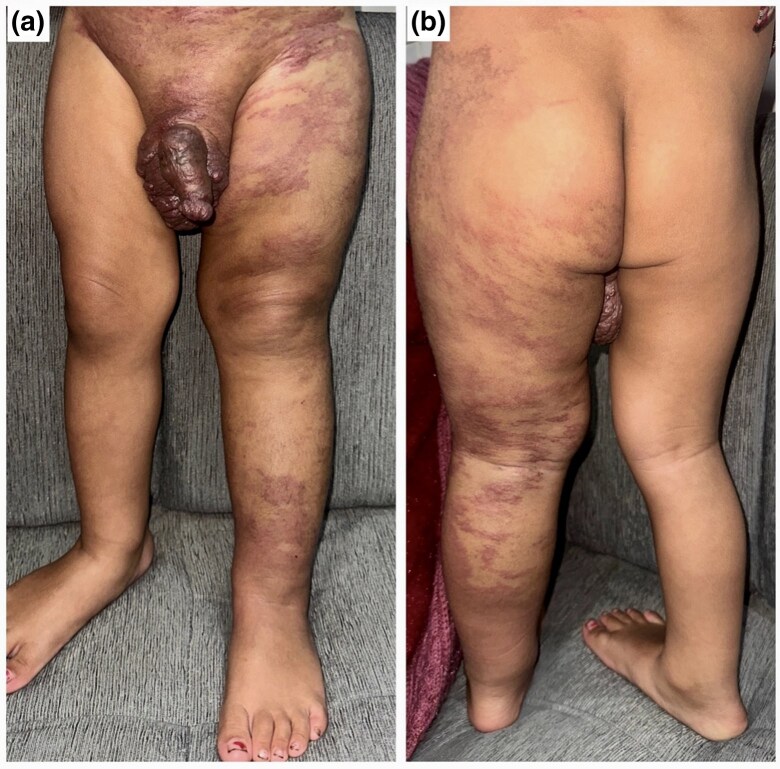
(a, b) Capillary malformation involving the left lower limb, lower abdomen, left lower back, suprapubic area and bilateral inguinal regions with penoscrotal asymmetry and nodularity; note the girth discrepancy (left lower limb longer and larger in circumference than the right limb).

**Figure 2:**
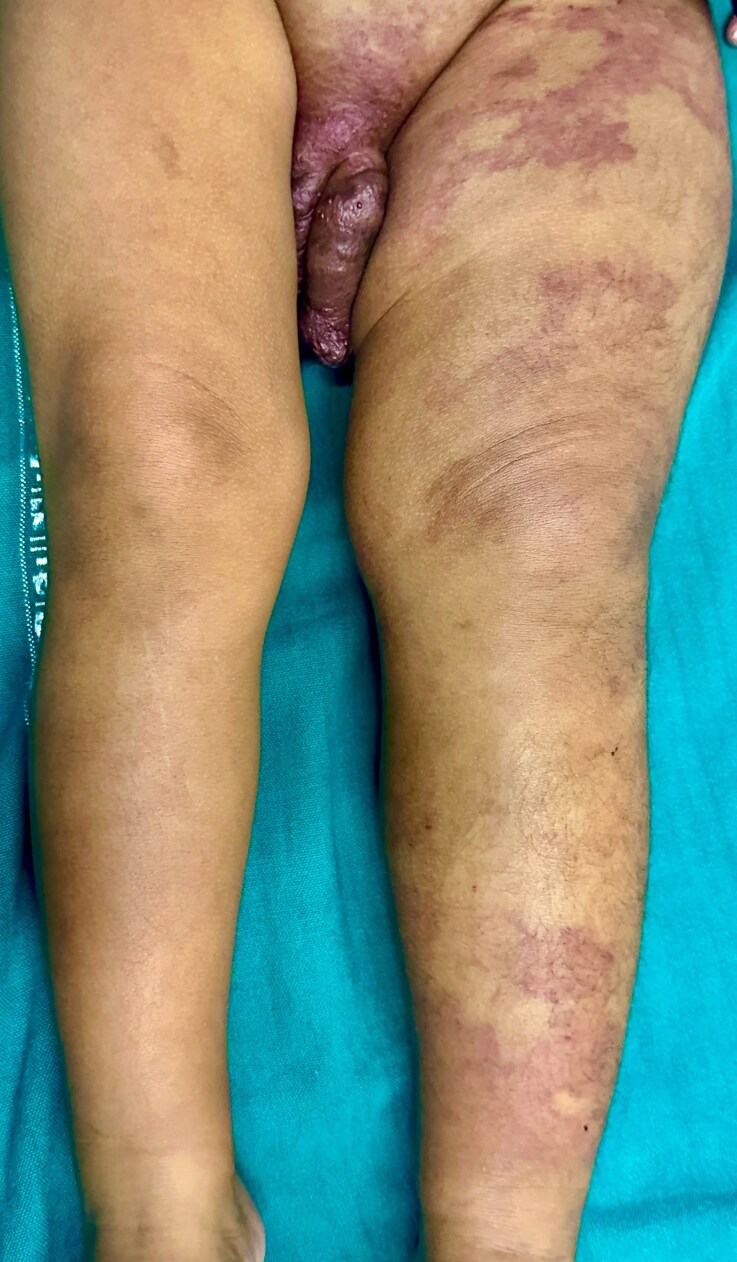
Supine position showing extensive capillary malformation; hypertrophy of the left lower limb is evident.

**Figure 3:**
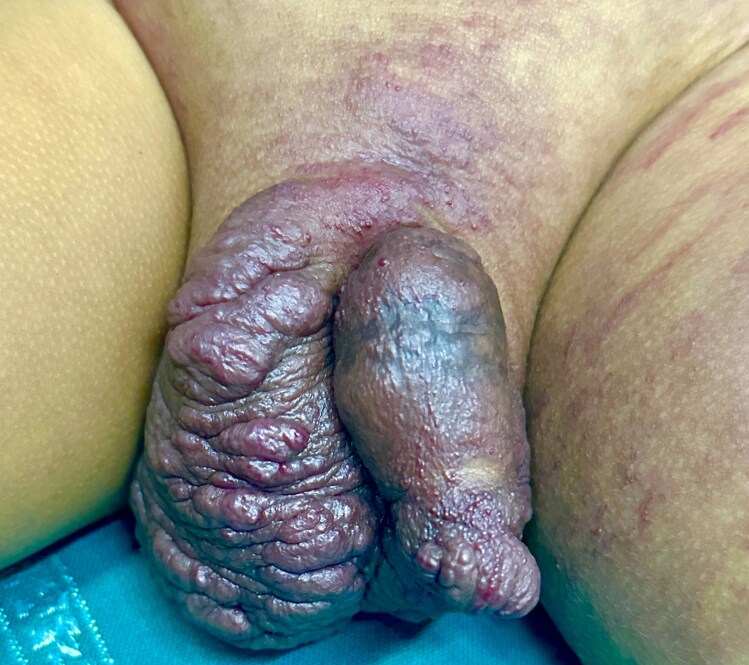
Involvement of the penoscrotal region. Penoscrotal skin is notably thickened, with exaggerated folds (rugae), and is densely covered with numerous raised, firm-appearing, violaceous papules, small nodules and angiokeratoma-like papules.

A 3-year-old boy presented with progressive swelling of the scrotum and left lower limb, noted since he was 6 days old. At 1 month, an erythematous, non blanchable patch developed on the left thigh and gradually extended to involve the lower limb, buttock, lumbar region, lower abdomen and bilateral inguinal areas. His parents also noticed progressive enlargement of the scrotum and penis, along with thickened, irregular skin. On examination, a large erythematous patch consistent with capillary malformation involving the left lower limb, lower trunk, suprapubic area and bilateral inguinal regions was present (Figure 1a, b). The left lower limb was longer and larger in circumference than the right limb (Figure 2). The scrotum and penis were asymmetrically enlarged, with violaceous papules and angiokeratoma-like papules (Figure 3).  The lesion was asymptomatic, with no signs of bleeding, systemic symptoms or visceral involvement. Both testes were palpable in the scrotum and were normally descended. On auscultation, there were no audible bruits over the lesion, and peripheral pulsation was normal.

**Figure 4:**
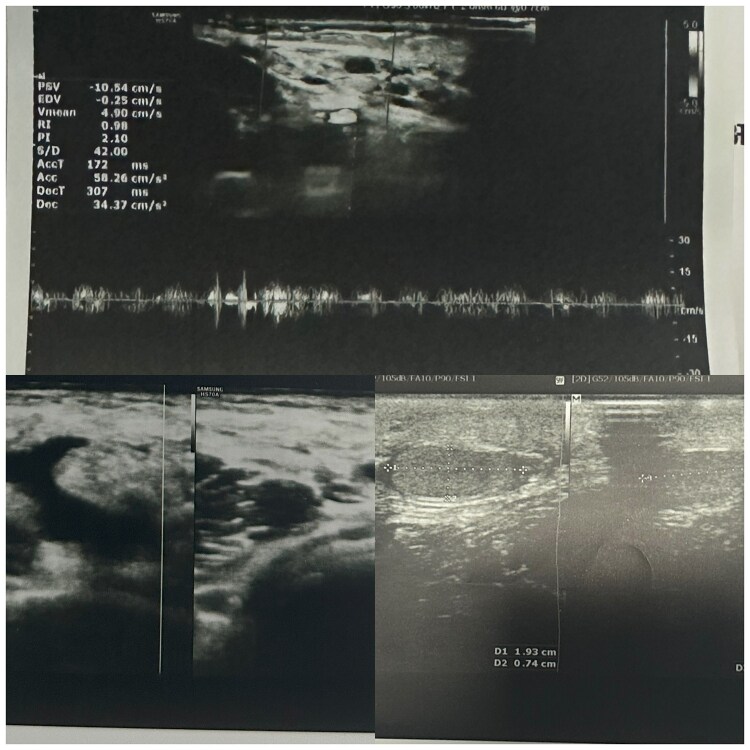
Scrotal ultrasound: heterogeneous subcutaneous lesions in the left thigh, lower abdominal wall and scrotum, with multiple cystic spaces showing venous flow.

Doppler ultrasonography showed heterogeneous subcutaneous lesions in the left thigh, lower abdominal wall and scrotum, with multiple cystic spaces demonstrating venous flow, suggesting a combined venolymphatic malformation (Figure 4). Routine laboratory investigations were normal, and computed tomography confirmed no visceral involvement. Magnetic resonance imaging (MRI) was not performed due to financial constraints. The patient was reviewed by urology, paediatrics, dermatology and radiology, with no recommended intervention at that time. The patient was managed conservatively, with advice on trauma prevention, physiotherapy to support mobility and regular follow-up. His parents were counselled regarding the potential risks of complications such as bleeding, thrombosis, ulceration and infection.

## Discussion

KTS was first described by Klippel and Trénaunay in 1900 and is associated with a somatic *PIK3CA* mutation, causing aberrant vascular growth and variable phenotypic expression.^1,3^ It is now recognized as part of the PIK3CA-related overgrowth spectrum (PROS).^3^ The triad of vascular malformation, limb hypertrophy and venous abnormality in our case highlights the classic yet complex features of KTS, with extensive cutaneous and penoscrotal involvement.^2^ Vicentini *et al*.^4^ noted penoscrotal vascular malformations in approximately 8.5% of cases.^4^ Similarly, Patel *et al*.^5^ observed genital involvement in about 17% of paediatric cases.^5^ In contrast, Husmann *et al*.^6^ in a series of 218 patients with KTS reported a higher incidence of 30% with urogenital involvement, including 7% with cutaneous genital abnormalities, underscoring the importance of careful evaluation for genitourinary involvement.^6^

Our case adds to the limited literature describing isolated penoscrotal involvement in early childhood without visceral disease, suggesting a less aggressive disease course. In contrast, abdominal, pelvic or perineal capillary malformations are associated with severe complications.^4^ Furthermore, simple KTS with blotchy capillary malformations, as seen in our patient, has a better prognosis than complex KTS, which typically presents with geographical lesions and higher complication risks.^2^ Diagnosis is typically clinical, with duplex ultrasound being the preferred test for assessing vascular anatomy and flow characteristics.^2^ High-resolution MRI is recommended in patients with cutaneous vascular malformations of the abdominal wall, pelvis or perineum, as these lesions may predict visceral genitourinary involvement, even in asymptomatic cases.^5^ Differential diagnosis for KTS includes Parkes Weber syndrome, neurofibromatosis type  I, Proteus syndrome, Sturge–Weber syndrome, Maffucci syndrome and Beckwith–Wiedemann syndrome.^1^ Currently, there is no definitive approved definitive treatment of KTS.^1^ The management of KTS is multidisciplinary, with a team-based approach, including dermatology, paediatrics, urology, vascular surgery, interventional radiology, orthopaedics, physiotherapy and psychological support.^3,6^ Genital involvement can lead to recurrent bleeding or haematuria, which are often difficult to control. Husmann *et al*.^6^ observed that compression and cauterization are effective in many cases, while refractory bleeding may require surgical excision. Haematuria may respond to conservative measures but sometimes necessitates cauterization, embolization or surgery.^6^ These findings highlight the importance of a stepwise approach in management. Management is primarily conservative, especially for genital lesions, focusing on minimizing complications.^4^ Careful surveillance is essential to detect and address complications such as bleeding, ulceration, deep vein thrombosis, pulmonary embolism, stasis dermatitis, cellulitis, infection and psychosocial consequences.^6,7^ Physiotherapy, trauma prevention, compression for limb involvement and parental counselling remain central to conservative care.^5^ Compression therapy has been reported as beneficial in managing varicose veins.^1^ Interventions such as laser or sclerotherapy can be considered for symptomatic capillary or lymphatic lesions, surgical correction may be required for deformities or varicose veins, and systemic therapy with sirolimus has been used in severe or refractory cases.^4,7^ In cases of extremity asymmetry and associated skeletal deformities such as scoliosis, surgical or orthopaedic management may be indicated. Capillary malformations are most effectively treated with pulsed dye laser therapy.^1^ Genital involvement in early childhood can have lasting effects on body image, social adjustment and future sexual health. Incorporating psychological counselling, parental guidance and structured long-term follow-up is essential to monitor urinary function, fertility and overall psychosocial well being.^5–7^

KTS is an uncommon vascular overgrowth disorder with variable clinical expressions.^1,4^ Genitourinary involvement in children is rare but carries important functional and psychosocial consequences, often requiring long-term monitoring.^3,6^ Our case demonstrates extensive penoscrotal disease without visceral extension, suggesting a less aggressive course. Such presentations expand the limited literature on paediatric KTS and underscore the importance of individualized, multidisciplinary management with attention paid to medical complications and psychosocial well being.^4,6,7^

## Data Availability

The data that support the findings of this study are available from the corresponding author upon reasonable request.
